# Data on chow, liver tissue and mitochondrial fatty acid compositions as well as mitochondrial proteome changes after feeding mice a western diet for 6–24 weeks

**DOI:** 10.1016/j.dib.2017.09.019

**Published:** 2017-09-18

**Authors:** Claudia Einer, Simon Hohenester, Ralf Wimmer, Lena Wottke, Renate Artmann, Sabine Schulz, Christian Gosmann, Alisha Simmons, Christin Leitzinger, Carola Eberhagen, Sabine Borchard, Sabine Schmitt, Stefanie M. Hauck, Christine von Toerne, Martin Jastroch, Ellen Walheim, Christian Rust, Alexander L. Gerbes, Bastian Popper, Doris Mayr, Max Schnurr, Angelika M. Vollmar, Gerald Denk, Hans Zischka

**Affiliations:** a Institute of Molecular Toxicology and Pharmacology, Helmholtz Center Munich, German Research Center for Environmental Health, 85764 Neuherberg, Germany; b Department of Medicine II - Grosshadern, Liver Center Munich, LMU Munich, 81377 Munich, Germany; c Institute of Toxicology and Environmental Hygiene, Technical University of Munich, 80802 Munich, Germany; d Research Unit Protein Science, Helmholtz Center Munich, German Research Center for Environmental Health, 85764 Neuherberg, Germany; e Institute of Diabetes and Obesity, Helmholtz Center Munich, German Research Center for Environmental Health, 85764 Neuherberg, Germany; f Department of Medicine I, Krankenhaus Barmherzige Brüder, 80639 Munich, Germany; g Biomedical Center, Department of Anatomy and Cell biology, LMU Munich, 82152 Planegg, Martinsried,, Germany; h Department of Pathology, LMU Munich, 80337 Munich, Germany; i Division of Clinical Pharmacology & Center for Integrated Protein Science Munich (CIPSM), Klinikum der Universität München, 80337 Munich, Germany; j Department of Pharmacy - Center for Drug Research, Pharmaceutical Biology, LMU Munich, 81377 Munich, Germany

## Abstract

The data presented in this article describe the fatty acid composition of chow, liver tissue and isolated liver mitochondria from mice fed for 6–24 weeks with a high caloric western diet (WD) in comparison to control diet (normal diet, ND). The fatty acid composition was measured via gas chromatography flame ionization detection (GC-FID). Moreover, WD-induced mitochondrial protein changes are presented in this work and were analyzed by mass spectrometry (LC–MS/MS). For further interpretation and discussion of the presented data please refer to the research article entitled “Mitochondrial adaptation in steatotic mice” (Einer et al., 2017) [1].

**Specifications Table**

**Table:** 

Subject area	Biochemistry, Molecular Biology
More specific subject area	Liver disease, Steatosis, Mitochondria
Type of data	text file, table
How data was acquired	Fatty acid detection was acquired by GC-FID (Carlo ERBA instruments HRGC 5300, Egelsbach, Germany) and proteomic data by LC–MS/MS (LTQ-Orbitrap XL, Thermo Scientific, Germany).
Data format	analyzed
Experimental factors	Lipid extraction according to the Folch method and protein digestion using trypsin.
Experimental features	Detection of fatty acid composition of normal diet (ND), western diet (WD) chow and of livers or isolated liver mitochondria from 6 to 24 weeks fed mice by GC-FID. Proteomic data of isolated liver mitochondria from ND and WD fed mice by mass spectrometry.
Data source location	Munich, Germany
Data accessibility Related research article	Data are available with this article. Einer et al. “Mitochondrial adaptation in steatotic mice”, Mitochondrion, 2017 (in press). [1]

**Value of the data**

• The data present the composition of fatty acids in normal diet (ND) and western diet chow (WD) in comparison to the fatty acid compositions of liver tissue and isolated liver mitochondria from ND and WD fed mice.
• The data provide a detailed molecular analysis of diet-induced mitochondrial protein and fatty acid changes.
• This data could be compared to human liver samples or data from other steatosis- and NASH-related animal models to reveal diet-induced adaptations and metabolic dysfunctions.

## 1 Data

The present data show the fatty acid composition of a western diet (WD) rich in saturated fat and enriched with fructose syrup in drinking water in comparison to standard rodent diet (normal diet, ND, Table 1). Male C57BL/6NCrl mice were fed with these diets for 6, 12 and 24 weeks and fatty acid compositions of liver tissue (Table 2) as well as from isolated liver mitochondria (Table 3) are displayed. Furthermore, a dataset of diet-induced mitochondrial protein changes is presented (Table 4).

**Table 1:** Fatty acid composition of the Western diet (WD) and Normal diet (ND) chow.

	**C10:0**	**C12:0**	**C14:0**	**C16:0**	**C16:1n9**	**C16:1n7**	**C17:0**	**C18:0**	
**ND** [mg/g]	n.d.	n.d.	**0.17**±0.14	**6.02**±1.88	**0.22**±0.10	**0.12**±0.11	**0.10**±0.04	**1.18**±0.45	
**WD** [mg/g]	**1.44**±0.54	**68.66**±4.67	**31.66**±0.90 ^⁎⁎⁎^	**18.08**±0.19 ^⁎⁎^	**0.04**±0.01	**0.04**±0.00	**0.13**±0.13	**13.89**±0.80 ^⁎⁎⁎^	
	**C18:1n9t**	**C18:1n9c**	**C18:1n7**	**C18:2n6c**	**C18:3n3**	**C20:0**	**C20:1n9**	**C20:3n3**	**total**

**ND** [mg/g]	n.d.	**7.09**±1.17	**0.55**±0.22	**16.97**±0.57	**1.94**±0.09	**0.12**±0.03	**0.20**±0.03	n.d.	**34.62**±4.13
**WD** [mg/g]	**0.16**±0.02	**8.49**±1.66	**0.24**±0.03	**6.22**±1.09 ^⁎⁎⁎^	**0.60**±0.01 ^⁎⁎^	**0.28**±0.06 ^⁎^	**0.09**±0.02 ^⁎^	**0.20**±0.00	**150.10**±3.72^⁎⁎⁎^

Gas chromatographic data of chow fatty acids (N=4). ^⁎^Significant to ND chow (Student's t test: ^⁎^ p < 0.05, ^⁎⁎^ p < 0.01, ^⁎⁎⁎^ p < 0.001, n.d. not detected).

**Table 2:** Fatty acid composition of mice livers fed a ND or WD.

	**6 weeks**		**12 weeks**		**24 weeks**	
	**ND** [µg/mg]	**WD** [µg/mg]	**ND** [µg/mg]	**WD** [µg/mg]	**ND** [µg/mg]	**WD** [µg/mg]
**C12:0**	0.13±0.03	0.45±0.26	0.11±0.08	0.52±0.07***	n.d.	n.d.
**C14:0**	0.10±0.01	0.83±0.36*	0.06±0.07	1.28±0.08***	0.11±0.03	1.30±0.18***
**C14:1n7c**	n.d.	n.d.	n.d.	0.08±0.06	n.d.	0.14±0.02
**C16:0**	5.84±0.26	8.30±1.96	6.70±1.88	15.37±1.55***	10.58±1.73	18.89 ±3.82*
**C16:1n9c**	0.09±0.01	0.19±0.10	0.14±0.11	0.69±0.22**	0.41±0.14	0.92±0.30*
**C16:1n7c**	0.47±0.02	1.51±0.64*	0.83±0.46	2.98±0.24	1.30±0.35	4.28±0.83**
**C17:0**	0.09±0.02	0.02±0.04*	0.07±0.05	0.09±0.02	0.12±0.02	0.07±0.00*
**C18:0**	3.90±0.30	3.90±0.45	3.66±1.01	4.63±1.08	3.83±0.53	3.18±0.31
**C18:1n9c**	4.03±0.37	7.35±2.05*	4.99±2.15	16.24±1.85***	11.12±3.24	22.35 ±4.78*
**C18:1n7c**	0.55±0.10	1.49±0.69	0.81±0.42	4.00±0.31***	2.06±0.82	7.11±1.23***
**C18:2n6c**	3.25±0.15	2.32±0.35**	3.16±1.08	2.05±0.48	5.35±0.75	2.60±0.31**
**C18:3n6c**	0.03±0.03	n.d.	0.02±0.05	0.03±0.05	0.08±0.00	n.d.
**C18:3n3c**	0.12±0.01	0.07±0.05	0.09±0.07	0.07±0.05	0.23±0.04	0.11±0.00**
**C20:0**	0.12±0.01	0.13±0.03	0.03±0.06	0.16±0.07*	0.14±0.02	0.13±0.03
**C20:1n9c**	0.09±0.01	0.16±0.05	0.09±0.08	0.49±0.06***	0.31±0.11	0.64±0.08**
**C20:2n6c**	n.d.	n.d.	n.d.	n.d.	0.05±0.01	0.29±0.02***
**C20:3n6c**	0.25±0.02	0.24±0.03	0.26±0.20	0.27±0.07	0.87±0.11	0.60±0.15*
**C20:4n6c**	2.23±0.19	1.91±0.32	1.58±0.39	1.65±0.52	3.67±0.61	2.30±0.32*
**C20:5n3c**	0.02±0.03	n.d.	0.69±1.32	n.d.	0.12±0.00	n.d.
**C24:0**	n.d.	n.d.	n.d.	n.d.	n.d.	0.19±0.07
**C22:6n3c**	0.92±0.07	0.70±0.18	0.74±0.22	0.58±0.17	1.82±0.39	0.89±0.12*
**Ratio** **18:0/16:0**	0.67±0.05	0.48±0.10*	0.55±0.10	0.30±0.05**	0.37±0.08	0.17±0.04*

Gas chromatographic dataset of liver tissue fatty acids after 6, 12 and 24 weeks feeding time (*N*=4). *Significant to ND control (Student's t test: * *p<*0.05, ** *p*<0.01, *** *p<*0.001, n.d. not detected).

**Table 3:** Mitochondrial fatty acid composition.

	**6 weeks**			**12 weeks**				**24 weeks**			
	**ND** [µg/mg]	**WD** [µg/mg]		**ND** [µg/mg]		**WD** [µg/mg]		**ND** [µg/mg]			**WD** [µg/mg]
**C16:0**	23.38±2.51	24.35±1.53		30.25±9.98		28.00±2.75			21.08±3.03		22.24±1.10
**C16:1n7c**	2.66±1.34	2.52±1.01		2.50±0.73		3.51±1.99			1.47±0.20		3.01±0.16***
**C18:0**	21.45±2.44	23.86±3.94		27.01±9.41		23.93±1.93			20.19±6.71		19.72±1.40
**C18:1n9c**	8.53±2.91	8.29±2.06		10.12±3.11		15.48±3.07*			6.40±1.00		11.67±1.80**
**C18:1n7c**	3.34±1.35	3.35±1.07		3.58±1.13		6.27±0.99*			2.52±0.37		5.55±0.58***
**C18:2n6c**	15.98±2.27	18.50±2.79		23.67±7.71		14.32±1.21			15.71±3.12		11.02±0.60
**C20:2n6c**	n.d.	n.d.		n.d.		1.96±0.58			n.d.		0.97±0.22
**C20:3n6c**	1.24±0.90	1.44±0.32		2.29±0.76		2.75±0.57			2.88±0.75		3.65±0.50
**C20:4n6c**	13.78±1.76	15.55±1.78		20.02±7.32		18.35±2.59			14.05±2.55		14.73±1.40
**C24:0**	n.d.	n.d.		n.d.		0.89±0.14			n.d.		0.95±0.25
**C22:6n3c**	4.15±0.63	4.96±0.72		6.58±2.63		5.32±0.78			5.53±1.04		4.70±0.69
**Ratio:**											
**unsat./sat.**	1.11±0.10	1.13±0.03		1.21±0.11		1.29±0.19			1.23±0.36		1.29±0.06
**16:1/16:0**	0.11±0.05	0.10±0.04		0.08±0.01		0.12±0.07			0.07±0.02		0.14±0.01***
**18:1/18:0**	0.40±0.11	0.35±0.06		0.38±0.03		0.65±0.14*			0.34±0.12		0.59±0.09*
**18:1/18:2**	0.77±0.35	0.65±0.25		0.59±0.09		1.51±0.20***			0.58±0.10		1.57±0.28**

Quantitative analyses from mitochondrial fatty acid methyl esters (*N*=4). *Significant to ND control (Student's *t* test: * *p*<0.05, ** *p<*0.01, *** *p*<0.001, n.d. not detected).

**Table 4:** Proteomic data from ND- and WD-mitochondria.

**WD vs. ND**	**fold enrichment**	**6 wk**	**12 wk**	**24 wk**
**Oxidative**	>1.5	Atp5d	mt-Co3	mt-Nd5, Ndufa11
**Phosphorylation**	<0.67		mt-Nd4, Uqcr11	
**Lipid degradation (ß-oxidation)**	>1.5	Acot13, Hadhb	Echs1, Eci2	Acot2, Cyp2d9, Ehhadh, Hadha
	<0.67	Acox2, Lipa, Phyh		Lipa, Phyh
**Lipid transport**	>1.5	Mttp, Slc27a5	Mcat	Scp2
**Lipid synthesis**	>1.5	Elovl2, Hmgcs2, **Tecr**		Echdc2, Elovl2, Hmgcs2, **Scd1**
**ER stress**	>1.5	Calr, Canx, Hsp90b1, Hspa5, P4hb, Pdia6		
**Lysosomal- /protein degradation**	>1.5 <0.67	Otub1 Ctsb, Lgmn, Lipa, Tpp1	Ctsb, Gaa, Gm2a has to go into > 1.5, Scpep1	Pmpca Ctsb, Gaa, Lipa, Fbxo16, Scpep1
**Autophagy/apoptosis**	>1.5	Pgrmc1, Pex14	Dap3	Nit1
	<0.67	Creg1, Nit1	Bnip3, Creg1, Pex14	
**Antioxidative**	>1.5	Sod1		P4hb
	<0.67	Gpx1	Gsta3, Gstm1, Gstz1	
**Steroid metabolism**	>1.5	Cyp2a12		Cyp2d9, Ugt2b36
	<0.67	Dhrs1	Cyp3a16, Dhrs1	Aldh1b1, Cyp3a16
**Translation**	>1.5	Eef1a1, Gm10036, Gm5619, Mrpl10, Mrpl43, Mrps23, Rplp0, Rplp2, Rps3	Rpl26, Rpl9	Gm10036, Mrps30, Rpl9
	<0.67		Mrrf, Rnaset2b	
**Retinol**	>1.5			Ugt2b36, Stra6
**Metabolism**	<0.67	Stra6	Cyp2a5, Cyp3a17, Stra6, Ttr	Cyp2a5, Cyp3a16
**Amino acid**	>1.5	Bhmt, Fmo5, Hadhb		Amt, Ehhadh, Hadha
**Metabolism**	<0.67	Ethe1, Nags	Fmo1, Gnmt, Nags, Qdpr	Ethe1, Gnmt, Sardh
**Miscellaneous**	>1.5	Aldob, Mettl7a1, Rpn2, Surf4, Tmem205, Ugt1a1, Vkorc1	Fmc1, H2-Q10, Hspe1, Selo	Atl2, Bdh1, Mcu, Mthfs, Nnt, Surf4, Ssr4
	<0.67	Mcu	Atl2, Iscu, Ptges2, Tmem256	Mup10, Oct
**Identified proteins**	Up	37 (6.5%)	11 (1.9%)	26 (4.6%)
(569)	Down	15 (2.6%)	28 (4.9%)	15 (2.6%)

Quantitative proteome comparisons of proteins that were either increased (>1.5-fold) or depleted (<0.67-fold) in WD vs. ND mitochondrial fractions (*N*=4).

## 2 Materials and methods

### 2.1 Animal studies and mitochondrial isolation

Male C57BL/6NCrl mice (Charles River, Sulzfeld, Germany) were housed according to the guidelines for the care and use of laboratory animals at the University Hospital Munich and had free access to water and chow. The mice received either a standard rodent diet (ND, V1535-0, Ssniff, Germany) or western diet (WD, 45% of calories from fat, Altromin Spezialfutter GmbH & Co. KG, Germany) enriched with 23.1 g/l fructose and 18.9 g/l glucose-monohydrate in drinking water for 6, 12 or 24 weeks [2].

Mitochondria from mouse livers were freshly prepared and purified by Percoll™ (GE Healthcare, Germany) as described earlier [3], [4].

### 2.2 Gas chromatographic fatty acid analysis

Fatty acids were extracted from either 250 µg purified mitochondria or 10 mg liver tissue according to the Folch method [5]. Cleared extracts were washed with 0.9%, NaCl and twice with methanol/water (1:1 v/v), the organic phase evaporated (N_2_), esterified and extracted (n-hexane). Fatty acid methyl esters were separated by capillary gas chromatography (BPX70 column from SGE, oven temperature 120–210 °C with increase of 2 °C/min, H_2_ as carrier gas, flame ionization detector) and identified by their retention time relative to standard mixtures (37 component FAME mix, Supelco, Germany). Quantification was done via C15:0 as internal standard and with Clarity Lite software (DataApex, Czech Republic).

### 2.3 Mitochondrial proteome analysis by mass spectrometry

#### 2.3.1 MS sample preparation

Mitochondria were lysed in 25 µl 2% SDS lysis buffer (2% SDS, 50 mM Tris-HCl, pH 7.4, 150 mM NaCl), heated up to 70 °C for 5 min and sonicated on ice six times for 15 s. Ten µg protein per replicate were diluted up to 400 µl in UA buffer (8 M urea in 0.1 M Tris/HCl pH 8.5) and digested applying a modified FASP procedure [6]. For cysteine reduction, 10 µl of 100 mM DTT were added to the samples and incubated for 30 min at 60 °C. After cooling, 10 µl of freshly prepared 300 mM iodoacetamide solution were added for 30 min at room temperature in the dark. Samples were centrifuged on a 30 kDa cut-off filter device (PALL) and washed thrice with UA buffer and twice with 50 mM ammoniumbicarbonate (ABC). Proteins were digested in 50 µl of 50 mM ABC for 2 h at room temperature using 1 µg Lys-C (Wako, Germany) and subsequently for 16 h at 37 °C using 2 µg trypsin (Promega, Germany). Samples were acidified with 0.5% trifluoroacetic acid (TFA) and analyzed on the OrbitrapXL as described.

#### 2.3.2 Mass spectrometry

LC–MS/MS analysis was performed as described previously on a LTQ-Orbitrap XL (Thermo Scientific, Germany) [7]. Briefly, samples were automatically injected and loaded onto the trap column and after 5 min, the peptides were eluted and separated on the analytical column separation by reversed phase chromatography operated on a nano-HPLC (Ultimate 3000, Dionex). A nano trap column was used (300 μm inner diameter×5 mm, packed with Acclaim PepMap100 C18, 5 μm, 100 Å, LC Packings, Sunnyvale, CA) before separation by reversed phase chromatography (PepMap, 25 cm, 75 µm ID, 2 µm/100 Å pore size, LC Packings) operated on a RSLC (Ultimate 3000, Dionex, Sunnyvale, CA) using a nonlinear 170 min LC-gradient from 5% to 31% of buffer B (98% acetonitrile) at 300 nl/min flow rate followed by a short gradient from 31% to 95% buffer B in 5 min and an equilibration for 15 min to starting conditions. From the MS prescan, the 10 most abundant peptide ions were selected for fragmentation in the linear ion trap if they exceeded an intensity of at least 200 counts and were at least doubly charged. During fragment analysis a high-resolution (60,000 full-width half maximum), the MS spectrum was acquired in the Orbitrap with a mass range from 300 to 1500 Da.

#### 2.3.3 Protein identification and label-free relative quantification

The RAW files (Thermo Scientific, Germany) were further analyzed using the Progenesis QI for proteomics (v1.0, Nonlinear Dynamics), as described previously [7], [8] with the following changes: Spectra were searched using the search engine Mascot (version 2.4, Matrix Science) against the Ensembl rat database (release 75; 25724 sequences). Search parameters used were: 10 ppm peptide mass tolerance and 0.6 Da fragment mass tolerance, one missed cleavage allowed, carbamidomethylation was set as fixed modification, methionine oxidation and asparagine or glutamine deamidation were allowed as variable modifications. A Mascot-integrated decoy database search using the Percolator algorithm calculated an average peptide false discovery rate of <1% when searches were performed with a Percolator score cut-off of 15 and a significance threshold of *p*<0.05. Peptide assignments were re-imported into Progenesis LC–MS. Normalized abundances of all unique peptides were summed up and allocated to the respective protein.

#### 2.3.4 Proteome data analysis

Data analysis was performed comparing ND and WD peptides in an age-matched manner and set as enriched or decreased if the mean raw abundance as well as the normalized abundance was ≥1.5 or ≤0.67 in WD compared to ND mitochondrial fractions, respectively. Using the protein and pathway databases UniProt [9], KEGG (Kanehisa Laboratories, Japanese) [10] and PANTHER [11], the identified proteins were grouped based to their cellular localization and metabolic pathway affiliation.

## References

[1] Einer C. (2017). Mitochondrial adaptation in steatotic mice. Mitochondrion.

[2] Tetri L.H. (2008). Severe NAFLD with hepatic necroinflammatory changes in mice fed trans fats and a high-fructose corn syrup equivalent. Am. J Physiol. Gastrointest. Liver Physiol..

[3] Schulz S. (2015). A protocol for the parallel isolation of intact mitochondria from rat liver, kidney, heart, and brain. Methods Mol. Biol..

[4] Schmitt S. (2014). Why to compare absolute numbers of mitochondria. Mitochondrion.

[5] Folch J., Lees M., Stanley G.H. Sloane (1957). A simple method for the isolation and purification of total lipides from animal tissues. J. Biol. Chem..

[6] Wisniewski J.R. (2009). Universal sample preparation method for proteome analysis. Nat. Methods.

[7] von Toerne C. (2013). Apoe, Mbl2, and Psp plasma protein levels correlate with diabetic phenotype in NZO mice - an optimized rapid workflow for SRM-based quantification. J. Proteome Res..

[8] Hauck S.M. (2010). Deciphering membrane-associated molecular processes in target tissue of autoimmune uveitis by label-free quantitative mass spectrometry. Mol. Cell. Proteom..

[9] A. Bateman, M.J. Martin, C. O'Donovan, M. Magrane, R. Apweiler, E. Alpi, R. Antunes, J. Arganiska, B. Bely, M. Bingley, C. Bonilla, R. Britto, B. Bursteinas, G. Chavali, E. Cibrian-Uhalte, AD. Silva, M. De Giorgi, T. Dogan, F. Fazzini, P. Gane, LG. Castro, P. Garmiri, E. Hatton-Ellis, R. Hieta, R. Huntley, D. Legge, W. Liu, J. Luo, A. MacDougall, P. Mutowo, A. Nightingale, S. Orchard, K. Pichler, D. Poggioli, S. Pundir, L. Pureza, G. Qi, S. Rosanoff, R. Saidi, T. Sawford, A. Shypitsyna, E. Turner, V. Volynkin, T. Wardell, X. Watkins, H. Zellner, A. Cowley, L. Figueira, W. Li, H. McWilliam, R. Lopez, I. Xenarios, L. Bougueleret, A. Bridge, S. Poux, N. Redaschi, L. Aimo, G. Argoud-Puy, A. Auchincloss, K. Axelsen, P. Bansal, D. Baratin, MC. Blatter, B. Boeckmann, J. Bolleman, E. Boutet, L. Breuza, C. Casal-Casas, E. de Castro, E. Coudert, B. Cuche, M. Doche, D Dornevil, S Duvaud, A. Estreicher, L. Famiglietti, M. Feuermann, E. Gasteiger, S. Gehant, V. Gerritsen, A Gos, N. Gruaz-Gumowski, U. Hinz, C. Hulo, F. Jungo, G. Keller, V. Lara, P. Lemercier, D. Lieberherr, T. Lombardot, X. Martin, P. Masson, A. Morgat, T. Neto, N. Nouspikel, S. Paesano, I. Pedruzzi, S. Pilbout, M. Pozzato, M. Pruess, C. Rivoire, B. Roechert, M. Schneider, C. Sigrist, K. Sonesson, S. Staehli, A. Stutz, S. Sundaram, M. Tognolli, L. Verbregue, AL. Veuthey, CH. Wu, CN. Arighi, L. Arminski, C. Chen, Y. Chen, JS. Garavelli, H. Huang, K. Laiho, P. McGarvey, DA. Natale, BE. Suzek, C. Vinayaka, Q. Wang, Y. Wang, LS. Yeh, MS. Yerramalla, J. Zhang., *UniProt: a hub for protein information.* Nucleic Acids Res, 2015. 43(Database issue): p. D204-12. UniProt: a hub for protein information, Nucleic Acids Res., 43 (Database issue), 2015, pp. D204–D212. 10.1093/nar/gku989 PMC4384041 25348405

[10] Kanehisa M. (2016). KEGG as a reference resource for gene and protein annotation. Nucleic Acids Res..

[11] Thomas P.D. (2003). PANTHER: a library of protein families and subfamilies indexed by function. Genome Res..

